# Everybody's Page

**Published:** 1889-11-16

**Authors:** 


### Everybody's Page.

Erysipelas is sometimes called St. Anthony's fire.

Professor Haddon brought some interesting anthropological specimens back from Torres Straits, and they are now on view in the British Museum.

One of the demonstrators in pathology at Cambridge University lately cut himself with a knife with which he had been dissecting a rabid dog. He immediately went to Paris to be treated by M. Pasteur.

An easy method of making albuminised milk for the sick-room is to shake together in a jar one pint of milk and the whites of two eggs until thoroughly mixed. Albuminised water may be flavoured with a few drops of lemon juice and two teaspoonsful of sugar.

The consumption of food per head in Victoria is in excess of that in America and Europe, and yet the climate of Australia requires that a man should eat less. The increase of hepatic and nervous diseases in Australia is thought to be due largely to the amount of meat consumed by its inhabitants. Thus it seems that the cheapness of food has its *cons* as well as its *pros*.

The precautions against leprosy taken by the chaplain of Robben Island have the merit of simplicity, and though he has been among the lepers for twelve years he has so far escaped contagion. He states that he puts on an old glove when he has to touch a door-handle of their ward, and when he comes from visiting them he washes his hands. He uses a separate chalice for these people when they attend Holy Communion.

In this age of hurry and worry, with its consequent nervous exhaustion, of which so much is now heard, the necessity of taking sufficient sleep cannot be insisted upon too forcibly. To lay down any hard and fast rule for its regulation is not possible, for, naturally, brain-workers require more than the drones of society; in fact, every brain-worker, if he wishes his powers to last, should take from eight to nine hours sleep out of every twenty-four. Charles Lamb did not think eight hours enough, wheras Sarah Bernhardt finds six hours a sufficient quantum of sleep.

The attention of the public has been called recently, by a series of letters in the daily papers, to the question of bread-baking. It is probably not generally known, or known as widely as is advisable for the public welfare, that a large number of bakers, even in the best districts of London, use ovens heated internally with bituminous coal. These ovens are frequently placed in underground cellars, and if the innocent consumers of the "staff of life" were aware of the filthy and the unwholesome conditions under which the so-called bread they eat is baked, their disgust would induce them to become their own bakers.

When M. Pasteur discovered the microbe of the silk-worm disease, he found that the germ of the microbe passed from the moth into the egg, and that, therefore, when the worm hatched out the microbe was in it, and this in time produced the disease. In order to stamp out this scourge, which was resulting most disastrously for the silk industry in France, M. Pasteur put each worm to lay its eggs on a separate piece of paper. Then he examined each moth carefully, and those he found diseased he burnt with the eggs they had laid, and only the eggs of the healthy moths were used. This plan proved so successful that in a short time the eggs from the station where M. Pasteur experimentalised, realised the sum of £1,100,000.

Why do Bryant and May use the ark as their trade mark? Because the animals went in in pairs, and therefore the ark must have been full of matches.

Electricity is being used in Italy to clarify wine. Fifty different sorts of wine have been experimented on, and they all stand travel better for the experiment.

The natives of Brazil cure their mosquito bites or any sort of "climate sores" with the root of a wild celery indigenous to Brazil, which they call "Aipa." They boil it down, and either use the water as a lotion or apply the leaves to the sore as a pad.

A pathetic account of cricket for the blind lately appeared in one of the morning papers. The players have a wicker ball with a bell in it, and are guided by ear only. One night, after dark, the blind played some friends who could see, and the latter were badly beaten.

We hear of an ingenious nurse who not long ago was attending a refractory old gentleman who refused to take his pill. She, determined not to be outdone, lodged the pill in the spout of a feeding-cup and filled the cup with beef-tea. Her ruse had the desired effect, for the unsuspecting patient, to her great delight, swallowed beef-tea and pill too. We do not recommend this plan, for it might result in a patient suffering the fate of the tenth little nigger.

It is proposed to include temperance teaching in our Government Educational Code, but be it remembered that temperance is not total abstinence. After the late startling figures regarding the short life of total abstainers, that doctrine is not likely to be taught by Government. Probably the short average life of teetotallers can be accounted for by the fact that those of weak physique, who cannot stand even a small quantity of alcohol, usually take the pledge.

Now that the importance of sanitation is becoming more universally recognised, the rival claims of the different methods of burying our dead are brought forward more prominently every day. Much attention has been paid to this question lately in America. A method which has for some time past been advocated on the other side of "the pond" is that of desiccation of the dead in closed chambers, built for the purpose. This method is now about to be put in practice by the New Mausoleum Company, of New York, in several of the most important cities of the States.

Dr. de Fournes, one of the contributors to the Paris *Journal d'Hygiene*, has a very poor opinion of English sobriety. "By nature," he says, "the Englishman drinks everything, and is always drinking. When he has not beer, porter, or pale ale, he drinks whisky or gin, and when the nerves of taste are badly demoralised, he falls back on ether and on chlorodyne, a drink the more dangerous in its present and future effects, that it remains the monopoly of well-to-do and aristocratic people, especially fashionable women." This is "a large order," as the Americans say, and though it is quite true that drunkenness is too common in England, it must be remembered that the anxiety felt by many to repress it causes it to be spoken of as a more general vice than it is. The vices of the fashionable world never belong to one country alone, and we doubt if a large proportion of fashionable women, either in England or any where else, indulge in the consumption of ether or chlorodyne. For the rest, we have our faults, and drunkenness is one of them; but, Dr. de Fournès, where do they drink absinthe most?

